# Irreversible blindness from hydrocephalus in recurrent pediatric pilocytic astrocytoma: a case report

**DOI:** 10.3389/fonc.2026.1800658

**Published:** 2026-04-22

**Authors:** Amro Hajja, Rasoul Turko, Lujain Fouad Khalaf, Roudina Amr Amin, Shady Salah Bagady, Luciana Aparecida Campos, Fouad Hasan Khalaf, Ovidiu Constantin Baltatu

**Affiliations:** 1 College of Medicine, Alfaisal University, Riyadh, Saudi Arabia; 2 Center of Innovation, Technology, and Education (CITE) at Anhembi Morumbi University, Anima Institute, Sao Jose dos Campos Technology Park, São José dos Campos, Brazil; 3 Al Iman General Hospital, Riyadh, Saudi Arabia

**Keywords:** blindness, hydrocephalus, intracranial hypertension, multidisciplinary management, optic atrophy, pediatric brain tumor, pilocytic astrocytoma, tumor recurrence

## Abstract

**Background:**

Pilocytic astrocytoma (PA) is the most common pediatric brain tumor, typically associated with favorable outcomes following surgical resection. However, irreversible vision loss due to elevated intracranial pressure (ICP) in posterior fossa PA is rare and often underrecognized. This case highlights the consequences of delayed detection of tumor recurrence and the critical importance of long-term surveillance in pediatric brain tumor survivors.

**Case Presentation:**

A 14-year-old male with a history of posterior fossa PA resection at age 4 presented with progressive headache, vomiting, and rapidly deteriorating vision after being lost to follow-up for 10 years. Neuroimaging revealed tumor recurrence with obstructive hydrocephalus. Imaging confirmed that the recurrent tumor was confined to the posterior fossa with no extension to or compression of the optic nerves, chiasm, or tracts. Despite emergency cerebrospinal fluid diversion and subtotal tumor resection followed by CyberKnife radiotherapy (45 Gy in 25 fractions), the patient developed permanent bilateral blindness. Fundoscopy confirmed severe bilateral optic disc atrophy most likely secondary to prolonged elevated ICP rather than direct tumor compression, although alternative contributing mechanisms could not be entirely excluded given the complex clinical course. At six-month follow-up, the patient remained clinically stable with no tumor progression but persistent vision loss and residual cerebellar dysfunction.

**Conclusion:**

This case demonstrates that posterior fossa PA recurrence can lead to irreversible blindness, most likely through sustained elevated ICP, even in the absence of direct optic pathway involvement. It underscores the necessity of systematic long-term surveillance protocols and prompt hydrocephalus management in pediatric brain tumor patients to prevent devastating but potentially avoidable complications.

## Introduction

Pilocytic astrocytoma is a low-grade glioma (WHO grade I) that primarily affects children and young adults (1). While generally considered less aggressive, the impact on vital functions can be profound, particularly when located in critical areas such as the optic pathways (2). The management of these tumors presents unique challenges, especially in young adults, where the tumor’s behavior and treatment outcomes may differ from pediatric cases (3). Vision loss associated with pilocytic astrocytoma can be attributed to multiple factors, including direct tumor effects and treatment-related complications, particularly radiation therapy (4), or a combination of both factors. Differentiating between these causes presents a significant diagnostic challenge, as both can manifest with similar clinical presentations and imaging features, and the onset and progression can vary (5).

The posterior fossa location of pilocytic astrocytoma presents distinct management challenges due to its anatomical proximity to critical structures and the potential for obstructive hydrocephalus. While direct tumor compression of anterior visual pathways occurs in supratentorial pilocytic astrocytomas, vision loss in posterior fossa tumors typically results from secondary mechanisms, including increased intracranial pressure (6). However, establishing that vision loss is predominantly ICP-mediated requires careful consideration of alternative etiologies, including peri-operative ischemic optic neuropathy and radiation-induced optic neuropathy, which presents a diagnostic challenge in complex cases with multiple interventions.

We present the case of a 14-year-old male with recurrent posterior fossa PA who developed permanent bilateral blindness secondary to prolonged elevated ICP from obstructive hydrocephalus, following a 10-year loss to follow-up after initial resection. This case highlights the critical importance of systematic surveillance protocols in pediatric brain tumor survivors and illustrates the devastating consequences of delayed recognition and management of tumor recurrence. It also illustrates the diagnostic complexity of attributing vision loss to a single mechanism in patients who undergo multiple interventions, including surgery and radiotherapy. This work has been reported in accordance with the CARE guidelines (7).

## Case presentation

Written informed consent was obtained from the patient’s father for the publication of this case report and any accompanying clinical details.

The clinical management of a 14-year-old male patient with recurrent pilocytic astrocytoma involved a series of critical events, interventions, and imaging assessments that are summarized in **Table 1**. The 14-year-old boy presented to the emergency department with a two-week history of unsteadiness, recurrent fainting, frequent falls, ear pain, progressive vision deterioration, and repeated episodes of right-sided focal seizures. He previously underwent resection of posterior fossa pilocytic astrocytoma at age 4. At that time, the patient had similar symptoms, requiring a midline suboccipital and infratentorial supracerebellar craniotomy for total tumor resection. Histopathological analysis confirmed a grade 1 pilocytic astrocytoma. Postoperatively, he recovered without fever, headaches, or vomiting, though cerebellar signs persisted. A pre-discharge CT (computed tomography) scan confirmed complete tumor resection. However, compensated communicating hydrocephalus was noted. The patient was discharged two weeks later in stable condition and scheduled for follow-up visits, but he did not return for his follow-ups.

**Table 1:** Sequence of events in the clinical management of the patient.

Event sequence	Clinical events	Imaging findings	Treatment/Intervention	Visual assessment
1. Initial Diagnosis (Age 4)	Diagnosed with pilocytic astrocytoma, midline suboccipital craniotomy for total tumor resection	Pre-discharge CT confirmed complete tumor resection; compensated communicating hydrocephalus noted	Total tumor resection performed. Follow-up appointments scheduled but not attended	Not documented
2. Presenting Symptoms (Age 14)	Two-week history of unsteadiness, recurrent fainting, frequent falls, ear pain, vision deterioration, right-sided focal seizures	CT scan revealed a heterogeneous mass in the posterior fossa causing moderate supratentorial hydrocephalus; no optic pathway compression (**Figure 1**)	Emergency placement of an external ventricular drain (EVD) via right parietal approach	Assessment 1 (Day of admission, before any intervention): RE — hand movements only; LE — finger counting at close range. Pupils sluggish but reactive
2.1. Surgical Intervention	Surgery for resection of the recurrent tumor via a transtentorial approach; approximately 4-hour procedure; patient hemodynamically stable throughout; no documented hypotension	MRI with contrast demonstrated a large recurrent mixed cystic and solid tumor with evidence of hemorrhage; tumor confined to posterior fossa (**Figure 2**)	Blood transfusions administered during surgery; subtotal tumor resection achieved	No immediate post-operative visual changes beyond pre-existing impairment
2.2. Postoperative Complications (3 days later)	Significant blood loss, intubated post-surgery	MRI showed moderate residual supratentorial hydrocephalus (**Figure 3**)	Emergent re-insertion of EVD due to accidental dislodgement; initiation of antibiotic therapy for suspected meningitis	—
2.3. Visual Assessment (one week after second resection)	Further visual deterioration, no light perception in both eyes	Fundoscopic examination revealed severe bilateral optic disc atrophy with hard exudates, consistent with chronic papilledema	Decision made to insert a ventriculoperitoneal (VP) shunt one week later	Assessment 2 (~1 week post-resection, PRIOR to VP shunt and PRIOR to radiotherapy): Bilateral no light perception. Severe bilateral optic disc atrophy confirmed
2.4. Multidisciplinary Discussion (including VP shunt insertion)	Case discussed in a multidisciplinary meeting; chemotherapy deemed unnecessary. VP shunt inserted under general anesthesia; favorable recovery. Patient discharged; experienced left focal seizure 2 days post-shunt; levetiracetam increased to 750 mg BID	Follow-up imaging suggested stable residual tumor and increased ventricular size	Scheduled follow-up MRI after two months to reassess treatment plan. VP shunt insertion performed. Evaluated in radiation oncology clinic ~1 month post-shunt; decision to proceed with upfront radiation therapy	Assessment 3 (~1 month after VP shunt, PRIOR to radiotherapy): Bilateral no light perception persisted
2.5. Radiation Therapy (6-month follow-up MRI after radiation completion)	Referred for radiation therapy following shunt placement. Complete bilateral blindness with confirmed optic disc atrophy had been established for several weeks before radiotherapy commenced	Follow-up MRI six months post-radiotherapy showed a stable residual mass but increased ventricular size (**Figure 4**)	Completed CyberKnife radiation therapy (45 Gy in 25 fractions)	Assessment 4 (6 months post-radiotherapy): Bilateral no light perception unchanged
2.6. Ongoing Management	Residual cerebellar dysfunction, ongoing seizure management	No new imaging findings; patient remains clinically stable	Ongoing follow-up appointments scheduled with neurosurgery and oncology	Ongoing monitoring: No change in visual function

Prior to this presentation, the patient had no significant medical issues and had an unremarkable social and family history. On admission, he was clinically stable, fully conscious, alert, and responsive to commands. Physical examination revealed bilateral horizontal nystagmus, fluent speech, and a broad-based gait, along with cerebellar signs, including dysmetria, a positive finger-to-nose test, a positive heel-to-shin test, and truncal ataxia. The remainder of the examination was unremarkable.

Visual Assessment 1 (Day of Admission, prior to any intervention): The patient underwent a visual assessment to evaluate his vision deterioration. On the right eye, he could only detect hand movements, while vision in the left eye was severely impaired, allowing him to count fingers only when held very close to his face. Both pupils were sluggish but reactive to light. No formal visual field testing or optical coherence tomography (OCT) was performed at this time.

**Figure 1:**
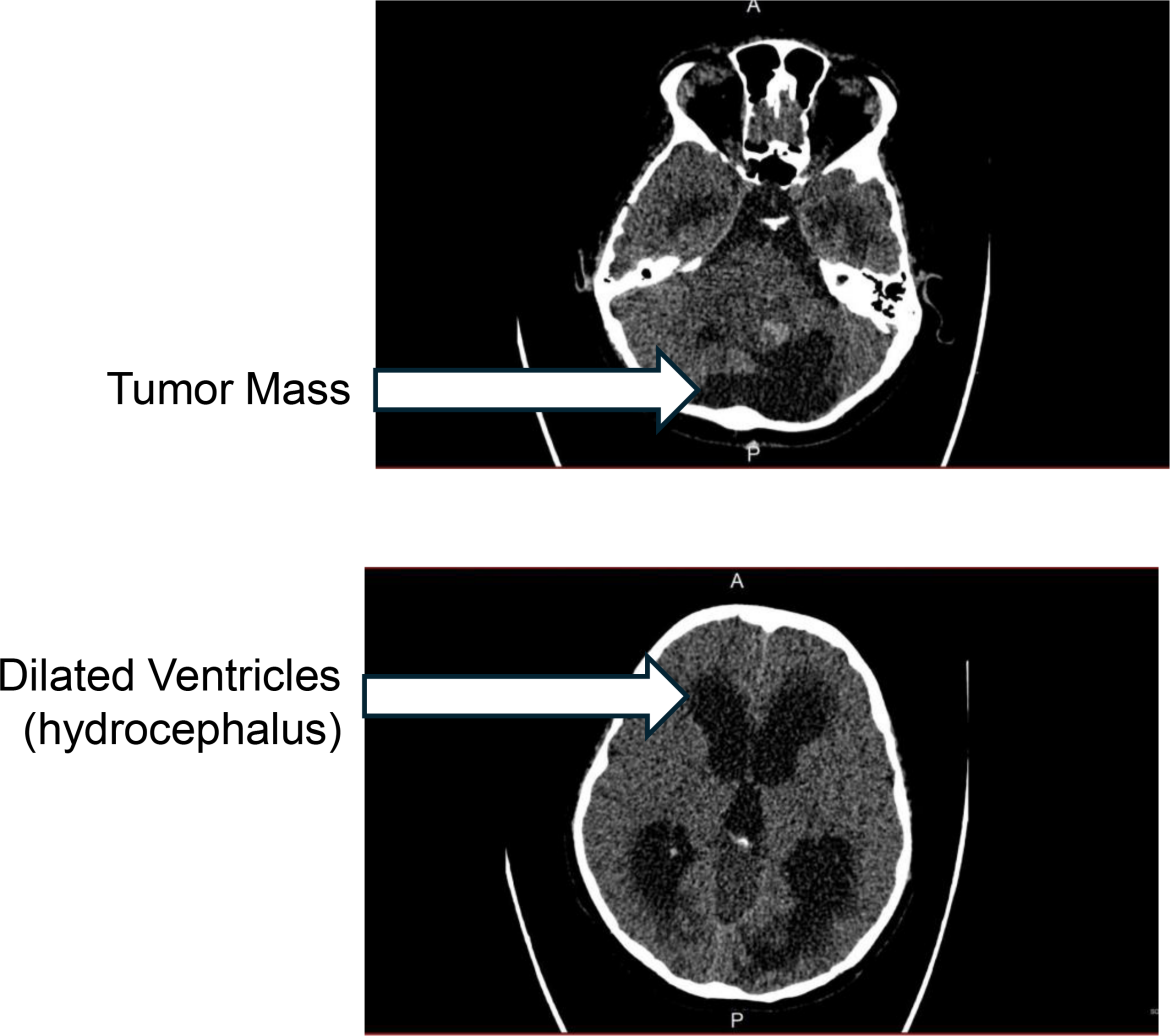
Non-contrast axial CT of the brain at presentation (age 14) demonstrating a posterior fossa mass occupying the fourth ventricle (arrow) with moderate supratentorial hydrocephalus (arrow). The mass is confined to the posterior fossa with no supratentorial extension or involvement of the anterior visual pathways. CT, computed tomography.

**Figure 2:**
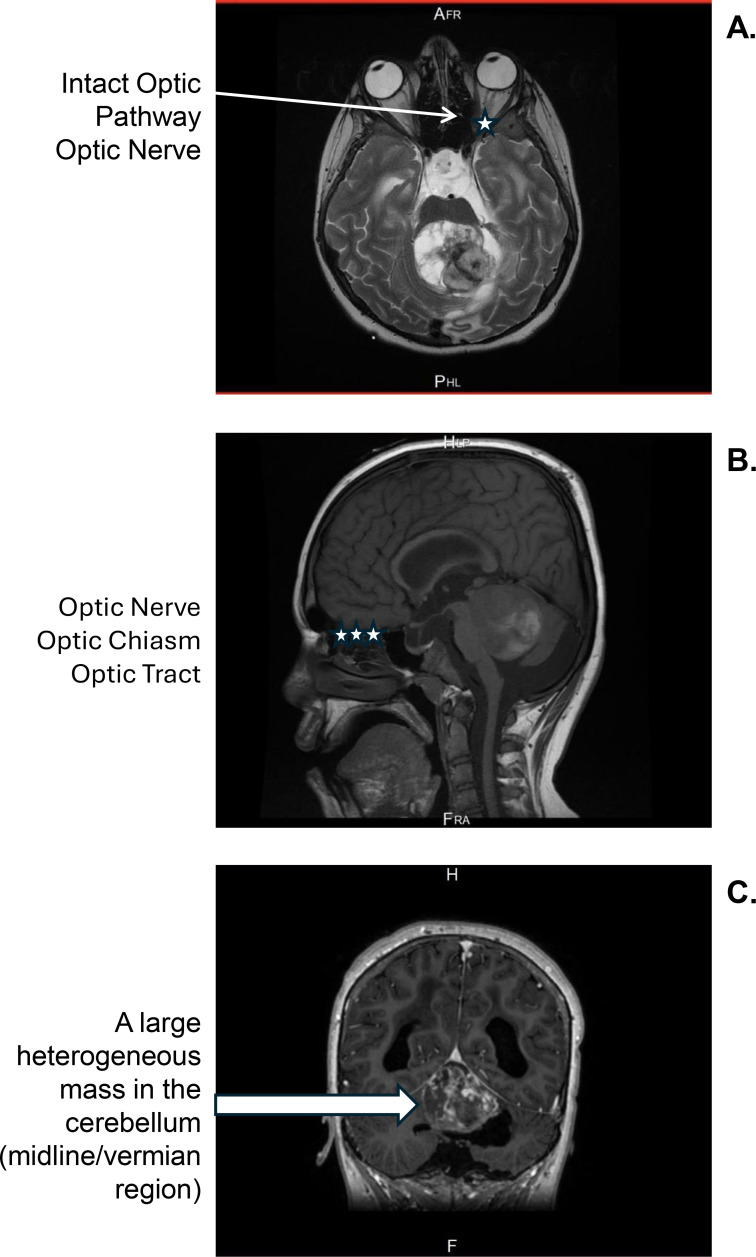
Pre-operative post-contrast MRI of the brain. **(A)** Axial view demonstrating the intact optic nerve (star) with no tumor involvement of the anterior visual pathway; arrow indicates the posterior fossa tumor. **(B)** Sagittal view with the anterior visual pathway structures identified (stars): optic nerve, optic chiasm, and optic tract. These structures appear intact with no evidence of tumor extension or compression, confirming the anatomical separation between the posterior fossa tumor and the anterior visual pathways. **(C)** Coronal view demonstrating a large heterogeneous mass in the cerebellum (arrow) with compression of the surrounding cerebellar tissue and the fourth ventricle, consistent with recurrent pilocytic astrocytoma causing obstructive hydrocephalus. MRI, magnetic resonance imaging.

A CT scan revealed the interval development of a heterogeneous mass in the posterior fossa, measuring 4.9 cm anteroposterior (AP) × 4.7 cm transverse (TVS) × 4.8 cm craniocaudal (CC), occupying the fourth ventricle and causing moderate supratentorial hydrocephalus. The CT scan demonstrated that the mass was confined to the posterior fossa, with no supratentorial extension, no mass effect on the optic nerves, chiasm, or optic tracts, and no evidence of leptomeningeal spread along the visual pathways (**Figure 1**). The patient underwent an expedited procedure for the placement of an external ventricular drain (EVD) using a right parietal approach. Subsequent MRI (magnetic resonance imaging) with contrast demonstrated a large recurrent mixed cystic and solid tumor in the posterior fossa, measuring 4.3 cm AP × 4.7 cm TVS × 4.5 cm CC, with evidence of tumoral hemorrhage. MRI confirmed that the tumor was limited to the fourth ventricle and superior cerebellar cistern (**Figure 2C**), with no involvement of or compression on the anterior visual pathways (**Figures 2A, B**). These findings were consistent with a recurrence of the pilocytic astrocytoma.

Three days after the initial diagnosis, the patient underwent surgery to excise the recurrent lesion through a transtentorial approach. The procedure lasted approximately 4 hours. Intraoperatively, significant blood loss occurred, with an INR (International Normalized Ratio) of 1.6 noted. Blood transfusions and tranexamic acid were administered to manage the bleeding. The patient remained hemodynamically stable throughout the procedure, with no documented episodes of hypotension. Despite these interventions, total tumor resection could not be achieved. Postoperatively, the patient was transferred to the ICU while intubated. Extubation was successfully performed on the second postoperative day, after which the patient was transferred to the ward. Postoperatively, the patient reported improvement in his headache. No immediate postoperative changes in visual function were noted beyond the pre-existing impairment documented on admission (Visual Assessment 1). However, during his ward stay, the EVD was accidentally dislodged by the patient, necessitating emergent reinsertion. He was also suspected to have developed meningitis and was started on vancomycin and meropenem. Shortly after, his creatinine levels became 166 µmol/L, prompting the discontinuation of vancomycin. Histopathological analysis of the resected lesion confirmed recurrent pilocytic astrocytoma.

**Figure 3:**
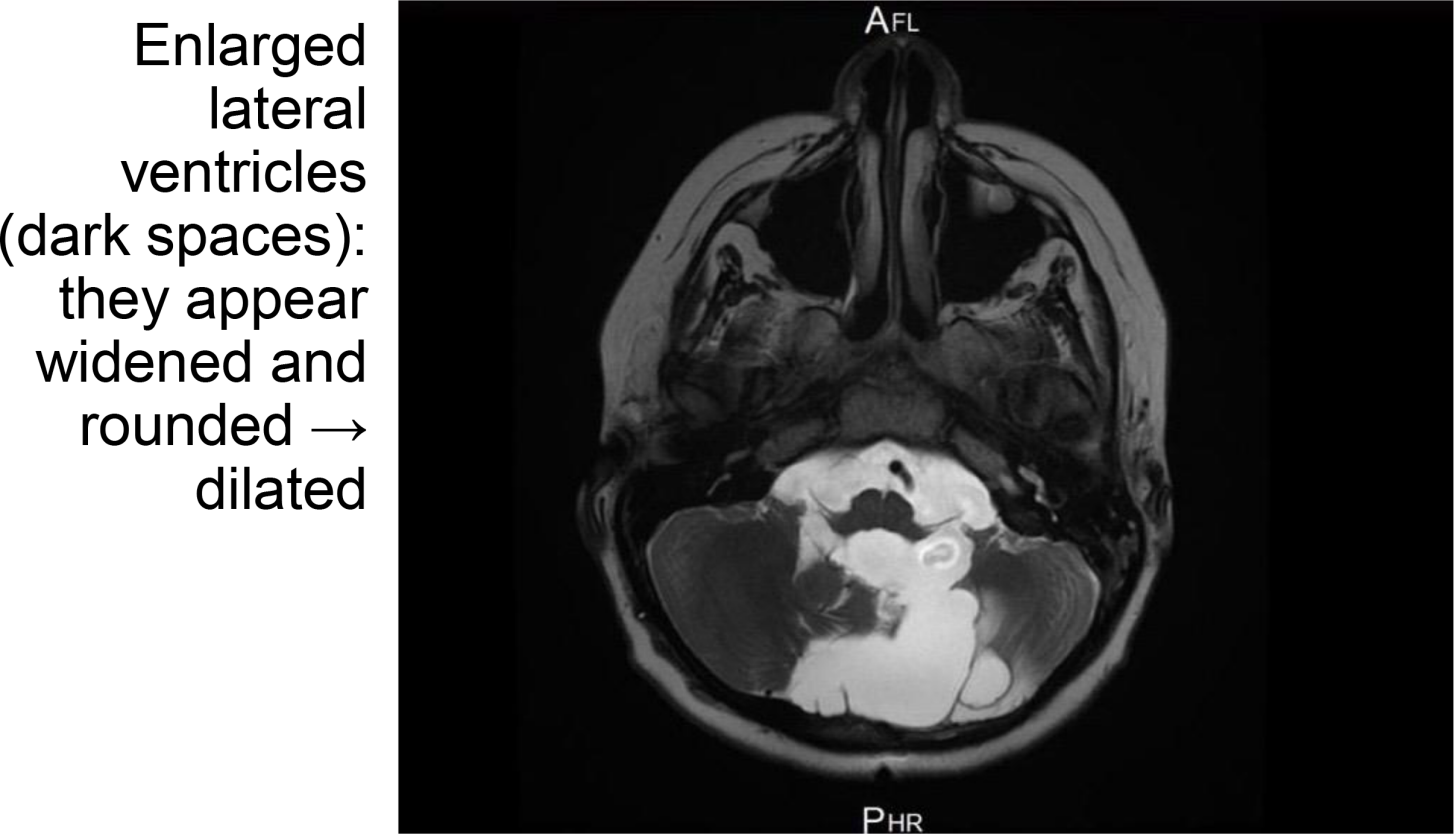
Post-operative axial T1-weighted MRI of the brain following subtotal tumor resection demonstrating persistent supratentorial hydrocephalus. The lateral ventricles appear widened and rounded, consistent with ongoing obstructive hydrocephalus despite surgical intervention. At this timepoint, Visual Assessment 2 confirmed bilateral no light perception with severe optic disc atrophy, prior to radiotherapy initiation. MRI, magnetic resonance imaging.

Subsequent MRI revealed moderate residual supratentorial hydrocephalus (**Figure 3**), with the residual mass measuring 4.2 cm AP × 4.9 cm TVS × 4.8 cm CC. The patient reported further vision deterioration, prompting a secondary visual assessment.

Visual Assessment 2 (approximately one week after tumor resection, prior to VP shunt insertion and prior to radiotherapy): This evaluation showed no light perception in both eyes, and dilated fundal examination revealed severe bilateral optic disc atrophy with hard exudates, most likely secondary to increased intracranial pressure. The fundoscopic findings were consistent with chronic papilledema progressing to secondary optic atrophy, rather than acute post-surgical changes, although other contributing factors could not be entirely excluded given the complex clinical course. Consequently, the decision was made to insert a ventriculoperitoneal shunt one week later.

The case was discussed in a multidisciplinary meeting, which concluded that the patient was not a candidate for chemotherapy but could potentially benefit from radiotherapy. The team decided to schedule a follow-up MRI after two months to reassess and finalize the treatment plan. The ventriculoperitoneal shunt was inserted under general anesthesia. The patient demonstrated favorable postoperative recovery, was extubated in the operating room, and transferred to the recovery unit for observation. He remained clinically, hemodynamically, and neurologically stable. The patient was subsequently discharged home with the following medications: acetaminophen 650 mg orally every 4 hours as needed for pain, levetiracetam 500 mg orally twice daily, and docusate 100 mg orally twice daily. Two days after the ventriculoperitoneal shunt insertion, the patient experienced a left focal seizure involving the face. The treatment plan was adjusted to increase levetiracetam to 750 mg orally twice daily, and the patient was discharged with this revised regimen.

One month later, the patient was evaluated in the radiation oncology clinic, where the management plan was discussed with his father. At this point, complete bilateral blindness (no light perception) with confirmed optic disc atrophy had been established for several weeks.

**Figure 4:**
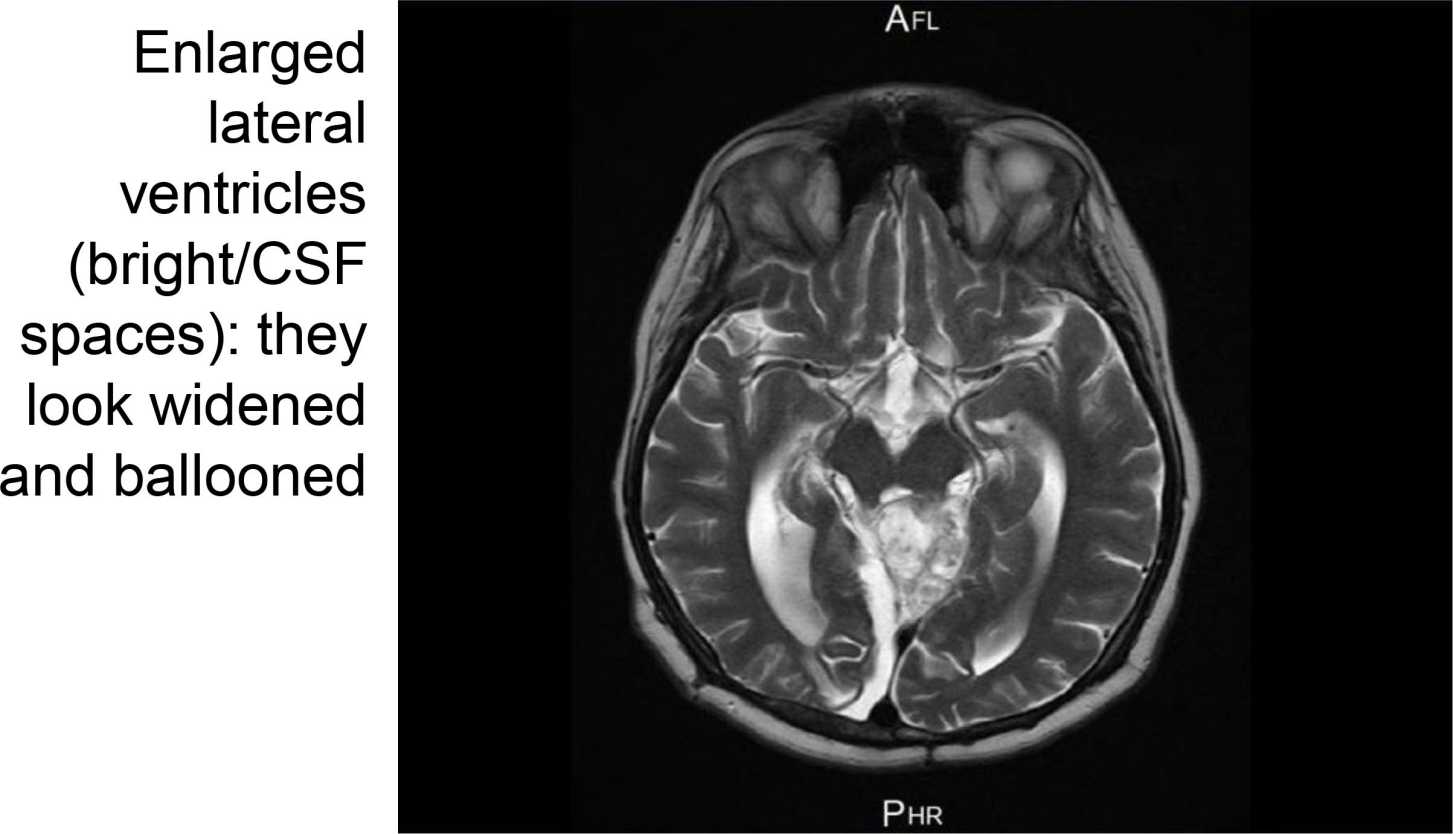
Six-month follow-up axial T2-weighted MRI of the brain after completion of CyberKnife radiotherapy demonstrating persistent supratentorial hydrocephalus with enlarged lateral ventricles. The persistent ventricular enlargement despite ventriculoperitoneal shunt placement illustrates the severity and chronicity of the obstructive hydrocephalus. Visual Assessment 4 confirmed persistent bilateral no light perception. MRI, magnetic resonance imaging.

Visual Assessment 3 (prior to radiotherapy initiation, approximately one month after VP shunt insertion): visual function remained unchanged, with no light perception bilaterally. It was decided to proceed with upfront radiation therapy. The patient successfully completed 45 Gray (Gy) in 25 fractions using the CyberKnife system. A follow-up MRI performed six months after the completion of radiotherapy revealed a stable residual multicystic, avidly enhancing mass in the superior cerebellar cistern. Notably, there was a marked interval increase in the size of the ventricular system, with enlarged lateral ventricles consistent with persistent hydrocephalus despite ventriculoperitoneal shunt placement (**Figure 4**), with a right frontal ventricular drain remaining in place.

Visual Assessment 4 (six-month follow-up after radiotherapy): Visual function remained unchanged, with persistent bilateral no light perception. To date, the patient continues regular follow-up in the clinic. He remains clinically stable and has not reported any new symptoms.

## Diagnostic challenges

Establishing the predominant mechanism of vision loss in this patient required systematic consideration of alternative etiologies. Several diagnostic challenges were encountered:

1. Differentiating ICP-mediated optic atrophy from direct tumor compression: Although the tumor was located in the posterior fossa, the possibility of occult leptomeningeal spread along the visual pathways required careful imaging review. CT and MRI at presentation confirmed no supratentorial extension or optic pathway involvement (**Figure 1**; **Figures 2A, B**), supporting an ICP-mediated mechanism.
2. Excluding peri-operative ischemic optic neuropathy (PION): The patient experienced significant intraoperative blood loss (INR 1.6) requiring transfusion during the approximately 4-hour procedure, which is a known risk factor for PION. However, the patient remained hemodynamically stable with no documented hypotension, no immediate postoperative visual changes were noted beyond pre-existing impairment, vision was already severely impaired prior to surgery (Visual Assessment 1), and the fundoscopic pattern was consistent with chronic papilledema rather than acute ischemic changes (see Discussion).
3. Excluding radiation-induced optic neuropathy (RION): Complete bilateral blindness with confirmed optic disc atrophy was documented prior to the initiation of CyberKnife radiotherapy (Visual Assessments 2 and 3), arguing strongly against RION as the cause of blindness (see Discussion).
4. Absence of advanced diagnostic tools: Formal visual field testing and OCT were not performed, which would have provided more detailed characterization of the optic nerve damage and its temporal progression. This limitation is acknowledged in the Discussion.

## Clinical management and multidisciplinary approach

The management of this complex case required coordinated care across multiple specialties. Initial emergency management focused on reducing intracranial pressure through external ventricular drainage, coordinated between neurosurgery and the critical care team. Ophthalmology provided crucial serial assessments of visual function, documenting the progression of optic atrophy despite intervention.

The neurosurgical approach involved tumor resection following cerebrospinal fluid (CSF) diversion, with neuroradiology guidance for surgical planning and post-operative monitoring. Following resection, the neuro-oncology team evaluated adjuvant therapy options, ultimately recommending CyberKnife radiation based on tumor characteristics and location.

Rehabilitation services, including vision rehabilitation specialists and neuropsychologists, played vital roles in the post-treatment phase. The neuropsychology team provided essential support for both patient and family in adapting to permanent vision loss, while physical therapy addressed residual cerebellar dysfunction.

Long-term follow-up protocols were established through collaboration between neurosurgery, neuro-oncology, and ophthalmology, with clear guidelines for imaging surveillance and functional assessments. This coordinated approach ensures comprehensive monitoring for potential tumor recurrence while addressing the patient’s ongoing rehabilitation needs.

## Discussion

### Summary of findings

This case describes a 14-year-old male with recurrent posterior fossa pilocytic astrocytoma (PA) who developed permanent bilateral blindness secondary to prolonged elevated intracranial pressure (ICP) from obstructive hydrocephalus, following a 10-year loss to follow-up after initial resection. Despite emergency cerebrospinal fluid (CSF) diversion and subtotal tumor resection followed by CyberKnife radiotherapy, vision could not be preserved. This case highlights a rare but devastating complication of pediatric posterior fossa tumors and underscores the critical importance of long-term surveillance in pediatric brain tumor survivors.

### Comparison with existing literature

Although PA is the most common pediatric brain tumor with generally favorable outcomes, blindness as a complication is exceedingly rare (1, 8). Vision loss in PA typically results from direct tumor compression of the optic pathways, radiation-induced optic neuropathy, or leptomeningeal spread (9, 10). Our case is notable because the clinical and imaging evidence suggests that blindness primarily resulted from sustained elevated ICP due to obstructive hydrocephalus, without direct involvement of the visual pathways.

Groves & Katz described a low-grade astrocytoma case where sudden monocular blindness resulted from a combination of direct tumor infiltration via leptomeningeal spread and chronic papilledema (9). Seth et al. reported subacute temporary vision loss in glioblastoma attributed to direct tumor mass effect, which resolved after treatment (11). In contrast, our patient’s blindness was irreversible and caused exclusively by prolonged ICP elevation from CSF obstruction, with fundoscopy confirming severe bilateral optic disc atrophy consistent with prolonged ICP elevation from CSF obstruction than direct compression (12, 13). This mechanism—complete permanent blindness from ICP alone in posterior fossa PA—has not been well-documented in the pediatric neuro-oncology literature. While ICP-mediated optic atrophy appears to be the predominant mechanism, the multifactorial nature of this patient’s clinical course warrants careful consideration of alternative contributing etiologies.

### Pathophysiological mechanism

The recurrent tumor occupied the fourth ventricle and superior cerebellar cistern, obstructing CSF flow and causing moderate supratentorial hydrocephalus. The prolonged interval between symptom onset and CSF diversion allowed sustained pressure on the optic nerves, leading to ischemia and irreversible optic nerve atrophy (13). Previous studies demonstrate that early CSF diversion in elevated ICP can correct papilledema and prevent progression to optic atrophy (14). Furthermore, microstructural compression of the optic nerve may improve within hours of ICP normalization, suggesting a narrow therapeutic window for vision preservation (15). In our patient, the ventriculoperitoneal shunt was inserted only after progressive vision deterioration had already occurred, likely beyond the window for reversibility.

### Consideration of alternative etiologies

In a complex case involving multiple interventions, it is essential to consider alternative mechanisms that may have contributed to the visual outcome.

Direct tumor compression of the visual pathways can be reasonably excluded based on imaging findings. CT and MRI at presentation demonstrated that the recurrent tumor was confined to the fourth ventricle and superior cerebellar cistern, with no supratentorial extension or involvement of the optic nerves, chiasm, or optic tracts.

Radiation-induced optic neuropathy (RION) is unlikely to have caused the blindness, as complete bilateral vision loss with confirmed optic disc atrophy was documented at Visual Assessment 2, approximately one week after tumor resection and prior to the initiation of CyberKnife radiotherapy, which commenced approximately one month later. Visual Assessment 3, performed immediately before radiotherapy initiation, confirmed persistent bilateral no light perception. The temporal sequence — with blindness fully established before any radiation exposure — argues strongly against RION as a causative factor. RION typically develops months to years after radiation therapy at doses exceeding 8 Gy in a single fraction or cumulative doses exceeding 50 Gy to the optic apparatus (4, 5). However, a potential contribution of radiotherapy to the inability to recover any residual visual function after shunting cannot be entirely excluded.

Peri-operative ischemic optic neuropathy (PION) represents a more nuanced consideration. The patient experienced significant intraoperative blood loss (INR 1.6) requiring transfusion during the approximately 4-hour procedure, which is a recognized risk factor for PION. However, several clinical findings argue against PION as the primary etiology: (1) vision was already severely impaired at presentation, before surgery (Visual Assessment 1: right eye — hand movements only; left eye — finger counting only); (2) the patient remained hemodynamically stable throughout the procedure, with no documented episodes of hypotension; (3) no immediate postoperative changes in visual function were noted beyond the pre-existing impairment; (4) PION typically presents as acute, painless vision loss in the immediate postoperative period, whereas this patient’s vision loss was progressive over weeks prior to surgery; (5) fundoscopy revealed changes consistent with chronic papilledema progressing to secondary optic atrophy (bilateral optic disc atrophy with hard exudates), rather than the acute disc pallor characteristic of PION; and (6) the bilateral and symmetric nature of the visual loss is more consistent with chronic ICP elevation than the typically unilateral or asymmetric presentation of PION. Nevertheless, a contributory role of peri-operative hemodynamic changes in accelerating the progression from severe visual impairment to complete blindness cannot be definitively ruled out.

Taken together, the evidence most strongly supports sustained elevated ICP as the predominant mechanism of vision loss in this patient. The progressive pre-operative visual decline, the imaging confirmation of tumor confinement to the posterior fossa without optic pathway involvement, the hemodynamic stability during surgery, the absence of immediate postoperative visual changes, and the fundoscopic findings consistent with chronic papilledema all point toward ICP-mediated optic atrophy as the primary etiology. However, we acknowledge that in a patient who underwent emergency surgery with significant blood loss followed by radiotherapy, a multifactorial contribution cannot be entirely excluded, and this represents a limitation inherent to complex clinical cases.

### Role of loss to follow-up

A critical factor in this case was the 10-year loss to follow-up after initial resection. While PA recurrence rates are relatively low following gross total resection, late recurrence can occur and necessitates long-term surveillance (16). Current guidelines recommend regular imaging for at least five years post-resection, with some centers advocating extended monitoring in pediatric cases (1, 2). Furthermore, long-term surveillance in pediatric brain tumor survivors is essential not only for detecting tumor recurrence but also for monitoring systemic consequences of the disease and its treatment. Our group has previously demonstrated that pediatric patients who underwent pinealectomy for brain tumors require long-term cardiovascular monitoring, as melatonin deficiency following surgery can lead to alterations in cardiac autonomic modulation and blood pressure regulation that persist years after treatment (17, 18). These findings reinforce the broader principle that pediatric brain tumor survivors require comprehensive, multidisciplinary follow-up programs that extend beyond tumor surveillance to include systemic health monitoring. Our case demonstrates that loss to follow-up in pediatric brain tumor survivors can result in delayed detection of recurrence and its complications, leading to preventable, irreversible neurological deficits. This emphasizes the need for robust follow-up systems, active family engagement, and clear communication about the importance of long-term surveillance.

### Clinical implications

This case has several implications for clinical practice:

1. Urgent ICP management: Papilledema in the setting of progressive headaches should prompt immediate neuroimaging and CSF pressure management, as the window for preventing permanent visual loss may be shorter than previously recognized (14, 15).
2. Systematic surveillance protocols: Pediatric brain tumor survivors require structured long-term follow-up programs with clear recall systems to minimize loss to follow-up, particularly in resource-limited settings (16).
3. Multidisciplinary coordination: Early involvement of neurosurgery, neuro-oncology, and ophthalmology is essential for timely recognition and management of complications in posterior fossa tumors (19, 20).
4. Family education: Active communication with families about the risks of recurrence and the importance of follow-up is vital for facilitating early detection and preventing irreversible sequelae.
5. Systematic consideration of alternative etiologies: In cases of vision loss associated with posterior fossa tumors, clinicians should systematically document the temporal relationship between visual decline and each intervention (surgery, radiation) to facilitate accurate etiological assessment. Serial visual assessments with specific timepoints relative to interventions are essential for distinguishing ICP-mediated damage from peri-operative or radiation-induced etiologies.

### Limitations

This case report has several limitations. First, as a single case, the findings cannot be generalized to all pediatric PA patients. Second, the 10-year gap in follow-up limits our understanding of the tumor’s progression and the timeline of hydrocephalus development. Third, formal visual field testing was not performed prior to the development of complete blindness, which would have provided more detailed documentation of visual function decline. Fourth, the absence of optical coherence tomography (OCT) imaging limits the characterization of optic nerve damage and prevents quantitative assessment of retinal nerve fiber layer thickness, which could have further supported differentiation between chronic papilledema and other etiologies. Fifth, while the clinical evidence most strongly supports ICP-mediated optic atrophy as the predominant mechanism — supported by the progressive pre-operative visual decline, hemodynamic stability during the 4-hour procedure, absence of intraoperative hypotension, and absence of immediate postoperative visual changes — the complex clinical course means that a minor contributory role of peri-operative or treatment-related factors in the final visual outcome cannot be definitively excluded. Sixth, the initial radiological images from the first surgery at age 4 were not available for comparison, which would have provided additional context regarding the baseline post-operative anatomy.

### Future directions

Future research should focus on establishing evidence-based surveillance protocols for pediatric brain tumor survivors, with particular attention to strategies for minimizing loss to follow-up. Studies examining the optimal timing of CSF diversion in posterior fossa tumors with hydrocephalus could help define the therapeutic window for vision preservation. Additionally, routine incorporation of OCT and formal visual field testing in follow-up protocols may enable earlier detection of subclinical optic nerve damage. Prospective studies documenting serial visual assessments with specific timepoints relative to surgical and radiotherapeutic interventions would provide stronger evidence for differentiating between ICP-mediated and treatment-related visual loss in posterior fossa tumors. Moreover, as demonstrated by our group’s experience with pinealectomized brain tumor survivors (17, 18), long-term follow-up programs should adopt a holistic approach encompassing not only oncologic surveillance but also cardiovascular, endocrine, and neurological monitoring, given the multisystem consequences of pediatric brain tumors and their treatment.

## Conclusion

This case demonstrates that recurrent posterior fossa pilocytic astrocytoma can lead to irreversible bilateral blindness, most likely through sustained elevated intracranial pressure from obstructive hydrocephalus, even in the absence of direct optic pathway involvement. While alternative contributing mechanisms — including peri-operative ischemic factors — cannot be entirely excluded, the progressive pre-operative visual decline, imaging findings, hemodynamic stability during surgery, absence of immediate postoperative visual changes, and fundoscopic pattern most strongly support ICP-mediated optic atrophy as the predominant cause. The 10-year loss to follow-up following initial resection resulted in delayed detection of tumor recurrence, ultimately leading to a potentially preventable, devastating visual outcome despite emergency intervention. This report underscores the critical importance of systematic long-term surveillance protocols in pediatric brain tumor survivors and emphasizes the need for prompt recognition and management of hydrocephalus to preserve vision. Enhanced follow-up systems, active family engagement, and clear communication about the risks of recurrence are essential to prevent similar outcomes in this vulnerable population.

### Key learning points

1. Predominant mechanism of blindness: Complete vision loss in posterior fossa pilocytic astrocytoma can result primarily from prolonged elevated intracranial pressure alone, without direct tumor compression of the optic pathways. However, in complex cases involving multiple interventions, a multifactorial contribution should be considered.
2. Critical role of surveillance: Loss to follow-up in pediatric brain tumor survivors can lead to delayed detection of recurrence and irreversible neurological complications; structured long-term follow-up programs are essential.
3. Narrow therapeutic window: The window for preventing permanent visual loss in elevated intracranial pressure may be shorter than previously recognized; urgent neuroimaging and cerebrospinal fluid diversion should be prioritized in patients with papilledema and progressive headaches.
4. Multidisciplinary coordination: Early involvement of neurosurgery, neuro-oncology, and ophthalmology is essential for timely recognition and management of complications in posterior fossa tumors.
5. Diagnostic complexity: Attributing vision loss to a single mechanism in patients with posterior fossa tumors who undergo multiple interventions requires careful consideration of the temporal sequence, imaging findings, and fundoscopic pattern. In this case, complete blindness was established prior to radiotherapy, the patient was hemodynamically stable during surgery with no immediate postoperative visual changes, and the progressive pre-surgical decline with chronic fundoscopic findings supported ICP-mediated damage as the predominant etiology.

## Data Availability

The original contributions presented in the study are included in the article/supplementary material. Further inquiries can be directed to the corresponding author/s.
